# The Effectiveness of Mindful Hypnotherapy on Psychological Inflexibility, Pain Acceptance, Headache Disability and Intensity in Females with Chronic Migraine Headache: A Randomized Clinical Trial

**DOI:** 10.3390/life13010131

**Published:** 2023-01-03

**Authors:** Hassan Khazraee, Maryam Bakhtiari, Amir Sam Kianimoghadam, Reza Hajmanouchehri

**Affiliations:** 1 Clinical Psychologist, Department of Clinical Psychology, School of Medicine, Shahid Beheshti University of Medical Sciences, Tehran, Iran; 2 Neurologist, Legal Medicine Research Center, Legal Medicine Organization, Tehran, Iran

**Keywords:** mindful hypnotherapy, chronic migraine, psychological inflexibility, pain acceptance, headache disability, headache intensity

## Abstract

This study was a randomized controlled design and examined the feasibility and effectiveness of mindful hypnotherapy on psychological inflexibility, pain acceptance, headache disability, and headache intensity in patients with chronic migraine headaches. The sample consisted of 38 females with chronic migraine who were randomly assigned to mindful hypnotherapy and medical treatment as usual groups. Psychological inflexibility pain scale (PIPS), chronic pain acceptance questionnaire-revised (CPAQ-R), headache disability inventory (HDI), diary scale for headache, and short-form McGill pain questionnaire 2 (SF-MPQ-2) were administered at baseline and post-treatment in both groups. The psychological inflexibility mean (SD) score was 81.00 (12.15) at baseline, which significantly decreased to 53.28 (17.06) after the intervention (*p* < 0.001). Additionally, the mean (SD) score of the pain acceptance was 46.44 (11.16), which significantly increased to 73.61 (15.65) in post-intervention (*p* < 0.001). Furthermore, the mean (SD) score of headache disability was 73.55 (19.48), which significantly decreased to 23.33 (19.88) in post-intervention (*p* < 0.001). Finally, headache intensity was 7.33 (0.98) and 5.78 (1.83), which significantly decreased to 2.77 (2.04), and 1.38 (1.48) after the intervention based on the Diary Scale for Headache and McGill Pain Questionnaire (SF-MPQ-2), respectively (*p* < 0.001). In conclusion, the results show that mindful hypnotherapy is a feasible and effective treatment for chronic migraine.

## 1. Introduction

Migraine is a common neurological disease, a highly debilitating disorder, so debilitating that it ranks second regarding years lived with a disability according to the World Health Organization and the Global Burden of Disease Study [1,2,3]. Moreover, migraine is the leading cause of disability worldwide in people under the age of 50, especially women, as this burden peaks between the ages of 35 to 39 years [1,4]. It is a chronic and often lifelong disease that is estimated to affect more than a billion people worldwide [1,3,4].

Chronic migraine (CM) is a migraine headache that occurs 15 or more days per month for more than 3 months and affects approximately 1.4–2.2% of the global population [5,6,7]. CM is associated with higher levels of disability and can have a profound impact on the quality of life and daily activities, leading to a substantial burden to the family and society [8,9,10].

One of the important key processes underlying the impact of headaches is psychological inflexibility [11], which includes the avoidance of pain and fusion with pain thoughts. The avoidance of pain, meaning attempts to control or limit contact with undesirable experiences such as headache pain, and avoiding headache triggers that decrease one’s ability to pursue a valuable living and may worsen the headache experience [11,12]. In contrast, psychological flexibility, which includes higher levels of acceptance of pain, and values-based action, are associated with less headache severity and disability in patients with migraine headaches [11]. Pain acceptance is a significant variance in headache impact [11], and there is growing evidence in migraine that supports the role of acceptance in headache severity and disability [13,14,15].

Additionally, other psychological factors, such as negative emotional states, can be related to the likelihood of attack onset, headache severity, performance, and treatment prognosis [16]. Many studies have shown that pain is associated with emotional dysregulation [17]. Specifically, headache patients have difficulty regulating anger [18]. Many headache patients turn their anger inward toward themselves, leading to the somatization of their emotions [19,20,21]. Furthermore, emotional awareness deficits with problems identifying and feeling emotions are strongly related to recurrent headaches [20].

In medical treatments, many pharmaceutical treatment options for migraine patients often result in side effects, poor tolerability, and limited effectiveness, which leads to high levels of unmet need, and a high number of patients report discontinuing or switching to alternative treatment options [22,23]. For example, simple analgesics or triptans are not sufficient to reduce the burden of the disease. These medications are ineffective in at least 30% of attacks, may be poorly tolerated, and, if overused, can even exacerbate migraine headaches [24]. Additionally, adherence to oral preventive anti-migraine treatments is low, with more than 60% of patients with chronic migraine abandoning them after two months. They are also ineffective in 40–50% of patients and poorly tolerated due to side effects [24,25].

In addition to pharmacological therapies, other treatment options exist, including psychological interventions [26]. Psychological interventions for migraine, such as cognitive-behavioral therapies, relaxation, and biofeedback, are effective in reducing the frequency of migraine attacks and disability [27,28,29,30,31]. However, not every person with migraine responds to these treatments [28], and they have some limitations [32,33], which require the development of new interventions.

The results of the review study by Hammond [34] has shown that clinical hypnosis is effective for treating headaches and migraine, along with being free from side effects and risks of adverse reactions, as well as the ongoing cost of the widely used medication treatments. It is also relatively brief and cost-effective. Another systematic review study by Flynn [35] demonstrates that hypnotherapy has a significant impact on the headache activity of patients with migraines and is statistically superior or equivalent to commonly used treatments. Clinical hypnosis can be used as a therapeutic supplement and a powerful tool for enhancing the performance of other psychotherapy [36]. Meta-analytic evidence suggests that cognitive-behavior therapy (CBT) combined with clinical hypnosis is a more effective form of psychotherapy than CBT alone. Furthermore, the effectiveness of psychodynamic psychotherapy increases with hypnosis [37,38].

Mindfulness-based treatments also represent an avenue to investigate effects in patients with chronic migraine [29]. The primary goals of mindfulness are a meditative lifestyle (i.e., open, nonjudgmental, “hear-and-now” awareness of reality), clinical behavior change, and health improvement of the person [39].

Mindful hypnotherapy (MH) tries to create a deeper level of change in patients by integrating mindfulness and hypnosis. This integration has critical clinical contemplation for improving intervention efficiency and effectiveness [40,41,42]. In mindful hypnotherapy, hypnotic suggestions increase the delivery of mindfulness principles (including nonjudgmental awareness and acceptance of internal experiences such as pain, difficult emotions, and thoughts, also compassion toward self and others, and finally resilience toward stressful situations and living based on values) that enables mindfulness to be absorbed and integrated more easily, efficiently, and effectively [40].

Despite considerable advances in treating migraine headaches, it remains the second most common cause of disability in the world [3]. It can lead to various disabling problems, such as psychological and emotional disorders, which can result in the relapse of headache symptoms. Considering the shortcomings of medical treatments for CM and the possible psychological causes of headaches, such as emotional regulation problems and avoidance of pain, it seems essential to use psychological interventions alongside pharmacological treatments. Therefore, chronic migraine should be managed by psycho-social-biological interdisciplinary treatments. It is assumed that psychological treatments, along with medical treatments, can increase therapeutic benefits and efficacy. Therefore, studying the effects of new psychological treatments, such as mindful hypnotherapy, seems necessary in patients with CM. MH may provide a unified approach to the treatment of both headache-related disability and pain. Thus, this study examines the feasibility and effectiveness of mindful hypnotherapy on psychological inflexibility, pain acceptance, headache disability, and headache intensity in patients with chronic migraine headaches.

## 2. Materials and Methods

### 2.1. Trial Design

This study is a randomized controlled design that was conducted as a doctoral dissertation in clinical psychology in 2022 at Shahid Beheshti University of Medical Science, Tehran, Iran. The Research Committee of Shahid Beheshti University of Medical Sciences (ethical code No: IR.SBMU.MSP.REC.1400.651) and the Iranian Registry of Clinical Trials (IRCT code No: IRCT20201009048974N2) have approved this study.

The assistant researcher, who was not aware of the study objective, generated the random number sequence using the random number generator function in SPSS. Then, the assistant researcher opened the envelope that was kept sealed, opaque, and numbered in sequence and then assigned the participants to the groups.

### 2.2. Participants

Participants included female adults with chronic migraine headaches without aura who were referred by an expert neurologist, Neurology Clinic, Moheb Mehr Hospital, Tehran, Iran. The sample size was calculated using G-Power software using an analysis of covariance considering effect size = 0.50 [43], a = 0.05, power = 0.85, and considering two-group resulted in 38 participants. Therefore, 38 participants based on the selection criteria listed below were selected by the convenience sampling method.

Inclusion criteria: the diagnosis of chronic migraine headaches without aura according to the International Headache Committee (IHS) and the Third International Classification of Headache Disorders (ICHD-3); a history of migraine for at least one year; having informed consent to participate in the study and sign a written consent form; age range of 18 to 50 years; availability for nine weekly sessions; in the case of receiving medications, the dosage and type of medication should be stable in the last months before the start of the study and remain stable during the study period.

Exclusion criteria: another chronic pain or headache diagnosis; having medication overuse headache (MOH); recent substance abuse; diagnostic indicators or a history of borderline personality disorder, bipolar disorder, psychosis, or schizophrenia due to contraindication with hypnosis; received psychological interventions in the last six months, or participated in another psychological intervention at the same time as the study.

### 2.3. Procedure

All participants provided informed consent to participate in the study and signed the written consent form. In addition to their right to withdraw to participate from the study, concerning the dropout rate from the study, a complete explanation was given regarding treatment conditions, the number of sessions, and randomization between treatment and control groups. Moreover, the participants have been thoroughly examined in terms of having enough time to attend the intervention sessions and the possibility of presence during the weekly seasons in the clinical psychology clinic in Taleghani hospital. Participants were randomly assigned to intervention (Mindful Hypnotherapy + Medical Treatment as Usual) and control (Medical Treatment as Usual) groups by randomization method. The intervention group consisted of nine weekly, 1-h sessions of mindful hypnotherapy. Participants in the MTAU group used the regular descriptive drugs and did not receive any psychological intervention during treatment but were given the opportunity to complete the intervention after participating in the study. The psychological inflexibility pain scale (PIPS), chronic pain acceptance questionnaire-revised (CPAQ-R), headache disability inventory (HDI), diary scale for headache, and short-form McGill pain questionnaire 2 (SF-MPQ-2) were conducted at baseline and post-intervention.

### 2.4. Intervention

The patients in the mindful hypnotherapy group were treated for nine weekly 1-h individual sessions of mindful hypnotherapy by using the mindful hypnotherapy protocol [40] adopted for headache patients. Major changes include an emphasis on nonjudgmental awareness with acceptance of pain in session 2. Session 3 is divided into 2 separate sessions, including mindfulness with nonjudgmental awareness of emotions and mindfulness with nonjudgmental awareness of thoughts with emphasis on headache-related thoughts before the headache starts and during the pain. Moreover, the therapist is a clinical psychologist with four years of experience in hypnotherapy and mindfulness-based interventions, in addition to working with patients with chronic headaches [44]. The intervention in every session included a didactic teaching component and hypnotic induction. In therapy sessions, treatment and suggestions were individualized to match the participant’s specific problems, needs, and goals, but home-practice audio was pre-recorded. The scripts were mostly based on mindful hypnotherapy: the basics for clinical practice manual [40]. The content of each session is as follows: (a) present-moment awareness, (b) nonjudgmental awareness of physical sensation with acceptance of pain, (c) nonjudgmental awareness of emotions, (d) nonjudgmental awareness of thoughts, (e) self-hypnosis, (f) compassion for self and others, (g) awareness of personal values and meaning in life, (h) integrated mindfulness, and (i) termination/transition to long-term practice [40]. At the end of each session, participants received daily practice audio recordings of their home exercises based on the content presented in that session.

### 2.5. Measures

Psychological Inflexibility in Pain Scale (PIPS): The PIPS is a 16-item scale used to assess the psychological inflexibility of people with chronic pain. The two main components of PIPS include avoidance of pain and fusion with painful thoughts. Items are rated on a 7-point Likert scale that ranges from 1 (never true) to 7 (always true). Higher scores meant greater levels of psychological inflexibility. The PIPS demonstrated good internal consistency as measured by Cronbach’s alpha, with 0.90, 0.75, and 0.89 for avoidance, fusion, and total scale, respectively [45].

Chronic pain acceptance questionnaire–Revised (CPAQ-R): The CPAQ-revised is a 20-item and rated on a 7-point scale from 0 (never true) to 6 (Always true). CPAQ-R has 2 subscales, including activity engagement (pursuit of life activities regardless of pain) and pain willingness (recognition that avoidance and control are often unworkable methods of adapting to chronic pain). A total score is calculated by summing all items, and higher scores indicate higher levels of acceptance. Internal consistency was reported with an alpha of 0.82 for activity engagement and 0.78 for pain willingness [46,47].

Headache Disability Inventory (HDI): HDI is a 25-item scale created by Jacobson et al. [48] for assessing different dimensions related to headache disability, including emotional and functional aspects. The three response options include no, sometimes, and yes, with 0, 2, and 4 scores, respectively. The total score ranged from 0 to 100 points, which ranks disability from absence to the maximum level [48]. Short-term stability was reported between 0.93 and 0.95 for one week, and long-term stability was reported between 0.76 and 0.83 for 2 months [48,49]. Cronbach’s alpha for emotional and functional aspects was reported as 0.68 and 0.83, respectively [50].

Diary Scale for Headache: This scale (38) was used to measure headache frequency and intensity. Patients record a diary of headache frequency and intensity on a rating scale from zero (absence of pain) to ten (most intense disabling headache) in a week. The mean headache intensity in one week was calculated by dividing the sum of severity scores by the number of headaches per week. The minimum headache intensity is zero, and the maximum score is ten. The reliability coefficient is estimated at 0.88 [51].

Short-form McGill pain questionnaire 2 (SF-MPQ-2): This questionnaire is used to measure the quality and intensity of the pain [52]. It consists of 22 different pain descriptors, and each item is rated based on a 0–10 scale with 0 (no pain) and 10 (the worst pain ever) during the past week. The total score is calculated by dividing the sum of the items by 22. SF-MPQ-2 comprises four subscales, including continuous, intermittent, neuropathic, and affective pain. Cronbach’s alpha coefficient for include continuous, intermittent, neuropathic, and affective pain and total scores were 0.87, 87, 0.83, 0.86, and 0.95, respectively [52].

### 2.6. Statistical Analysis

Collected data were analyzed with SPSS 24 software. The chi-square test was used to compare the demographics of the two groups (see Table 1). Moreover, Independent Samples *t*-Tests were used to identify baseline differences between the intervention and control groups in clinical characteristics (see Table 2). Furthermore, Levene’s Test of Equality of Variances (see Table 2) was used to describe the normality of our variables and followed by parametric tests. Table 3, Table 4 and Table 5 provide the mean scores and standard deviation (SD) of the dependent variables. Analysis of covariance (ANCOVA) was performed to determine the difference between the means of intervention and control groups, with controlling pre-intervention scores for measures of psychological inflexibility, pain acceptance, headache disability, and headache intensity. A *p*-value less than 0.05 was considered significant in all tests.

## 3. Results

Of the 38 females with chronic migraine who had been randomly assigned to intervention and control groups (19 in each group), 34 (18 in the intervention group and 16 in the MTAU control group) completed the post-test, and their data were included in the final analysis. Those who did not complete the intervention were excluded from the study.

Table 1 demonstrates the demographic characteristics of the participants. The result of the chi-square test shows that there were no statistically significant differences between the study groups in demographic variables (*p* > 0.05). The Independent *t*-Test (see Table 2) also shows that there were no statistically significant differences between groups at baseline in clinical characteristics (*p* > 0.05). The results of Levene’s test of equality of variances (see Table 2) also revealed that all variables, including psychological inflexibility, pain acceptance, headache disability, and headache intensity in both intervention and control groups were normally distributed (*p* > 0.05).

Table 3, Table 4, Table 5, Table 6 and Table 7 show the participants’ mean scores and the results of the analysis of covariance for dependent variables in the baseline measures and after the intervention. According to the presented data in Table 3, there was a clinically significant reduction in the avoidance of pain and fusion with pain thought subscales, in addition to the total psychological inflexibility in the mindful hypnotherapy group compared with the MTAU group (*p* < 0.001). The effect size for the avoidance of pain, fusion with pain thought, and total psychological inflexibility subscales were 0.53, 0.33, and 0.48, respectively.

The total scores of pain acceptance and its subscales, including pain willingness and activity engagement, have a clinically significant improvement in the mindful hypnotherapy group compared with the MTAU group (*p* < 0.001), with the effect size of 0.66, 0.44, and 0.65, respectively (see Table 4).

Table 5 indicates that emotional, functional, and total scores of headache disability have a significant reduction in the intervention group from the baseline measure to the post-test; in fact, the reduction was not observed in the MTAU group (*p* < 0.001). The effect size for emotional, functional, and total scores were 0.70, 0.62, and 0.67, respectively.

Finally, headache frequency and headache intensity based on the diary scale for headache (see Table 6) have a clinically significant reduction in the mindful hypnotherapy group compared with the MTAU group, with an effect size of 0.67 and 0.60 (*p* < 0.001). Furthermore, the result of the McGill pain questionnaire (see Table 7) shows that the total pain intensity, in addition to the subscales, including continuous pain, intermittent pain, neuropathic pain, and affective pain has a clinically significant reduction in the intervention group compared with the MTAU group (*p* < 0.001). The effect size for total pain intensity was 0.62, and for continuous, intermittent, neuropathic, and affective pain was 0.72, 0.45, 0.41, and 0.67, respectively.

## 4. Discussion

This study examines the feasibility and efficacy of mindful hypnotherapy, a novel intervention that incorporates elements of both mindfulness and hypnosis for treating headache disability and intensity alongside improving psychological inflexibility and pain acceptance in females with chronic migraine. Results from severe migraine patients showed statistically significant changes in outcome variables after treatment, including improvements in psychological inflexibility and pain acceptance compared with control groups. Furthermore, the mindful hypnotherapy group had a statistically significant decrease in headache disability and headache intensity after treatment compared with the control group.

These findings were similar to those of a previous study of mindful hypnotherapy. The results of the study by Olendzki et al. [43] show that MH showed clinically significant improvements in overall mindfulness and psychological flexibility in college students. Furthermore, the results of the study by Slonena and Elkins [53], a brief (three sessions) mindful hypnosis intervention compared with the active control group, show that MH is an effective intervention for increasing mindfulness skills and reducing stress reactivity. Additionally, in clinical hypnotherapy, the result of the review study by Hammond [34] and Flynn [35] has shown that clinical hypnosis is effective in treating headaches and migraine.

The effectiveness of mindful hypnotherapy can be explained by the mechanisms underlying migraine headaches and related components and processes of the treatment. One of the most important treatment principles is mindfulness. Manipulating attention with mindfulness techniques can affect the affective and sensory aspects of pain perception. Also, nonjudgmental awareness of bodily sensation with acceptance increases body awareness, and parasympathetic activity leads to improved body mechanisms and reduces pain [54,55]. Mindfulness improves cognitive and affective regulation of nociceptive input by a reappraisal of sensory and pain perception in a nonjudgmental manner that leads to decreased nociception [56,57,58]. Moreover, the avoidance of pain and fusion with pain thoughts as psychological inflexibility is an underlying factor in the impacts of headaches [11,12], and high levels of acceptance of pain are associated with reduced headache severity and disability [11,13,14,15]. Also, the determination of values and living a life based on values when pain is present leads to altered responses to pain and reduced disability [15]. Thus, in mindful hypnotherapy, a new relationship between bodily sensation and pain has been created by nonjudgmental awareness and acceptance of pain as a physical sensation and active willingness to have pain and respond to pain-related experiences without unnecessary and unhelpful struggles for pain control or attempts to avoid them. Furthermore, hypnotic induction techniques, as a powerful tool to create cognitive, perceptual, physical, and physiological changes, have been used [59]. In a hypnotic state, the patient is more receptive to new ideas in their mental, physiological, emotional, and behavioral frameworks [60].

Moreover, both mindfulness and hypnosis have neuroplasticity effects [40,61,62,63]. The result of a study by Jiang et al. [64] indicates that during a hypnotic state, reduced activity in the dorsal anterior cingulate cortex (dACC) increased the functional connectivity between the dorsolateral prefrontal cortex (DLPFC) and the executive control network (ECN) and the insula in the salience network (SN), also reduced connectivity between the ECN (DLPFC) and the default mode network (DMN) activity and posterior cingulate cortex (PCC). As a result, these areas of neural activity underlie focused attention and enhanced somatic and emotional control [40,64]. Thus, hypnosis induction in mindful hypnotherapy could be a change in neural activity that contributes to attention and emotional control, two important factors that contribute to headaches. However, research is needed to further elucidate the biological mechanisms of MH.

Based on this study, mindful hypnotherapy is recommended as a feasible and effective psychotherapy for improving psychological inflexibility, pain acceptance, headache disability, and headache intensity in patients with chronic migraine headaches. These results also support the potential utility of the MH model for chronic headaches. Mindful hypnotherapy can be combined with other medical treatments that are helpful to this population. This approach provides a promising basis for further therapy development and challenges us to reconsider the pain experience.

The main limitation of this study was the gender of the study sample. The participants were female, and this led to the limited interpretation and extrapolation of results to other genders. Future investigations should attempt to replicate and extend these results and tests of generalizability to another gender. Moreover, another limitation of the study is that the results of the study rely on subjective measures. Other limitations included the lack of an active control condition and a relatively small sample size. Therefore, further research could be conducted with an active control group and a larger sample size. Additionally, future research is needed to examine the impact of mindful hypnotherapy delivery in a group format. Both mindfulness and hypnotherapy can be performed in a group context and could be very beneficial, enabling many individuals to be treated at the same time. Research is also needed to investigate the feasibility, efficacy, and effectiveness of MH for chronic tension-type headaches, other chronic pain conditions, irritable bowel syndrome, and psychological issues in chronic conditions such as cancer and MS.

## 5. Conclusions

The results of the current study show that mindful hypnotherapy is promising as being an effective intervention for improving psychological inflexibility, pain acceptance, headache disability, and headache intensity in females with chronic migraine. Given that the results in psychological inflexibility and pain acceptance were conducted in both mindfulness and hypnosis contexts, this novel intervention shows the potential for being a unique and valuable contribution to chronic migraine interventions. However, because of the subjective measures, it is unclear if the subjective improvement that we found is down to the design of the study or to the treatment under investigation. In conclusion, this study suggests that mindful hypnotherapy is a very effective way of alleviating the symptoms of headache, and given the promising findings for MH, neurologists and psychotherapists may wish to give serious consideration to mindfulness in combination with hypnosis as a treatment option when working with patients with headache.

## Figures and Tables

**Table 1:** Demographic Characteristics of the participants.

Variable		MH Mean (SD)	MTAU Mean (SD)	Independent Samples *t*-Test/Chi-Square
Age		33.67 (7.38)	36.56 (8.24)	0.288
Time since the first onset of headache (years)		12.56 (7.73)	13.44 (7.64)	0.741
Headache frequency (days in last month) ^a^		17.44 (3.69)	19.00 (5.21)	0.319
Gender		All Female	All Female	
Education Level	Diploma	4	4	0.904
	Associate	1	1	
	Bachelor	5	5	
	Master	7	4	
	PhD	1	2	
Marital Status	Single	8	2	0.121
	Married	9	13	
	Divorced	1	1	
Occupation	Employee	5	6	0.078
	Housewife	5	9	
	Student	5	1	
	Free Job	3	0	
Family history of headache	With family history	15	11	0.317
	Without family history	3	5	
Medication	Antidepressants	8	9	0.922
	Anticonvulsants	4	3	
	Beta-blockers	3	2	
	Other Medications	3	2	

^a^ Reported number of headaches in the one month before commencing the study. Abbreviations: MH = Mindful Hypnotherapy; MTAU = Medical Treatment as Usual; SD = Standard Deviation.

**Table 2:** Levene’s Test of Equality of Variances and Independent *t*-Test for Equality of Means.

Variable	Subscales	Levene’s Test of Equality of Variances		Independent *t*-Test for Equality of Means in Pretreatment
		F	Sig.	Sig. (2-Tailed)
Psychological Inflexibility	Avoidance of pain	1.937	0.174	0.195
	Fusion with Pain Thoughts	2.72	0.109	0.662
	Total	2.232	0.145	0.219
Pain Acceptance	Pain Willingness	0.729	0.399	0.881
	Activity engagement	2.804	0.104	0.429
	Total	2.349	0.135	0.526
headache disability	Emotional	2.316	0.137	0.799
	Functional	2.146	0.153	0.710
	Total	2.814	0.103	0.744
Diary Scale for Headache	Headache frequency in a week	3.327	0.077	0.898
	Headache intensity	2.313	0.138	0.758
MPQ	Continuous	0.791	0.381	0.368
	Intermittent	0.281	0.600	0.215
	Neuropathic	0.235	0.631	0.211
	Affective	0.181	0.673	0.802
	total	1.296	0.263	0.448

**Table 3:** Comparison of Outcome Measures at Pretreatment to Post-treatment for psychological Inflexibility.

Variable	Group	Mean (SD)		ANCOVA			
		Pre	Post	F	*p*-Value	Effect Size	Observed Power
Avoidance of Pain	MH	44.83 (10.32)	26.17 (11.48)	35.15	0.000 ***	0.531	1.000
	MTAU	49.18 (8.65)	45.75 (9.55)				
Fusion with Pain Thoughts	MH	36.16 (3.56)	27.11 (7.04)	15.38	0.000 ***	0.332	0.967
	MTAU	36.81(4.94)	35.50 (5.47)				
Total scores	MH	81.00 (12.15)	53.28 (17.06)	29.47	0.000 ***	0.487	1.000
	MTAU	86.00 (10.99)	81.25 (13.36)				

Note: Covariate includes pre-intervention scores. *** *p* < 0.001.

**Table 4:** Comparison of Outcome Measures at Pretreatment to Post-treatment for Pain Acceptance.

Variable	Group	Mean (SD)		ANCOVA			
		Pre	Post	F	Sig	Effect Size	Observed Power
Pain Willingness	MH	18.50 (6.44)	26.94 (7.92)	24.86	0.000 ***	0.445	0.998
	MTAU	18.13 (7.99)	16.87 (6.50)				
Activity Engagement	MH	27.94 (8.35)	46.67 (10.22)	58.07	0.000 ***	0.652	1.000
	MTAU	25.69 (8.01)	24.19 (10.53)				
Total	MH	46.44 (11.16)	73.61 (15.65)	61.10	0.000 ***	0.663	1.000
	MTAU	43.81 (12.78)	41.06 (13.26)				

Note: Covariate includes pre-intervention scores. *** *p* < 0.001.

**Table 5:** Comparison of Outcome Measures at Pretreatment to Post-treatment for headache disability.

Variable	Group	Mean (SD)		ANCOVA			
		Pre	Post	F	Sig	Effect Size	Observed Power
Emotional	MH	39.22 (10.36)	12.11 (10.11)	72.85	0.000 ***	0.701	1.000
	MTAU	38.37 (8.77)	37.00 (8.23)				
Functional	MH	34.33 (9.53)	11.22 (10.17)	51.98	0.000 ***	0.626	1.000
	MTAU	33.13 (9.20)	33.00 (9.12)				
Total scores	MH	73.55 (19.48)	23.33 (19.88)	64.72	0.000 ***	0.676	1.000
	MTAU	71.50 (16.67)	70.00 (16.76)				

Note: Covariate includes pre-intervention scores. *** *p* < 0.001.

**Table 6:** Comparison of Outcome Measures at Pretreatment to Post-treatment for headache intensity based on Diary Scale for Headache.

Variable	Group	Mean (SD)		ANCOVA			
		Pre	Post	F	Sig	Effect Size	Observed Power
Headache frequency in a week	MH	4.89 (1.18)	1.55 (1.24)	62.89	0.000 ***	0.670	1.000
	MTAU	4.94 (0.99)	4.56 (0.89)				
Headache intensity	MH	7.33 (0.98)	2.77 (2.04)	48.30	0.000 ***	0.609	1.000
	MTAU	7.43 (0.82)	6.97 (1.30)				

Note: Covariate includes pre-intervention scores. *** *p* < 0.001.

**Table 7:** Comparison of Outcome Measures at Pretreatment to Post-treatment for headache intensity based on McGill pain questionnaire.

Variable	Group	Mean (SD)		ANCOVA			
		Pre	Post	F	Sig	Effect Size	Observed Power
Continuous	MH	7.12 (1.71)	1.87 (1.84)	81.32	0.000 ***	0.724	1.000
	MTAU	7.60 (1.23)	7.33 (1.52)				
Intermittent	MH	5.59 (2.41)	1.36 (1.50)	26.24	0.000 ***	0.458	0.998
	MTAU	4.53 (2.47)	4.05 (2.51)				
Neuropathic	MH	3.73 (2.34)	0.86 (1.20)	21.91	0.000 ***	0.674	1.000
	MTAU	2.69 (2.36)	2.72 (2.22)				
Affective	MH	7.11 (1.60)	1.44 (1.79)	64.15	0.000 ***	0.674	1.000
	MTAU	6.96 (1.67)	6.59 (1.91)				
Total	MH	5.78 (1.83)	1.38 (1.48)	51.91	0.000 ***	0.626	1.000
	MTAU	5.31 (1.69)	5.04 (1.77)				

Note: Covariate includes pre-intervention scores. *** *p* < 0.001.

## Data Availability

Data are available on request from the authors by email.
